# Integrated cellular network of transcription regulations and protein-protein interactions

**DOI:** 10.1186/1752-0509-4-20

**Published:** 2010-03-08

**Authors:** Yu-Chao Wang, Bor-Sen Chen

**Affiliations:** 1 Laboratary of Control and Systems Biology, Department of Electrical Engineering, National Tsing Hua University, Hsinchu, 30013, Taiwan

## Abstract

**Background:**

With the accumulation of increasing omics data, a key goal of systems biology is to construct networks at different cellular levels to investigate cellular machinery of the cell. However, there is currently no satisfactory method to construct an integrated cellular network that combines the gene regulatory network and the signaling regulatory pathway.

**Results:**

In this study, we integrated different kinds of omics data and developed a systematic method to construct the integrated cellular network based on coupling dynamic models and statistical assessments. The proposed method was applied to *S. cerevisiae *stress responses, elucidating the stress response mechanism of the yeast. From the resulting integrated cellular network under hyperosmotic stress, the highly connected hubs which are functionally relevant to the stress response were identified. Beyond hyperosmotic stress, the integrated network under heat shock and oxidative stress were also constructed and the crosstalks of these networks were analyzed, specifying the significance of some transcription factors to serve as the decision-making devices at the center of the bow-tie structure and the crucial role for rapid adaptation scheme to respond to stress. In addition, the predictive power of the proposed method was also demonstrated.

**Conclusions:**

We successfully construct the integrated cellular network which is validated by literature evidences. The integration of transcription regulations and protein-protein interactions gives more insight into the actual biological network and is more predictive than those without integration. The method is shown to be powerful and flexible and can be used under different conditions and for different species. The coupling dynamic models of the whole integrated cellular network are very useful for theoretical analyses and for further experiments in the fields of network biology and synthetic biology.

## Background

With the advance of technologies (whole genome sequencing, expression profiling, and other high-throughput experiments), vast amounts of data, which cover different aspects of cellular physiology, have emerged [1,2]. These kinds of 'omics' data, which include the genome sequencing data (genomics), microarray-based genome-wide expression profiles (transcriptomics), protein abundances data (proteomics), etc., provide unprecedented views of cellular components in the biological systems [1]. However, the challenge for current researchers lies in how to interpret these large-scale data sets and extract true information to understand biological systems more thoroughly. With the amount of data accumulated, it is appropriate to understand living organisms from a system point of view. Computational techniques, which are able to combine these large and heterogeneous data sets, will provide us useful tools to gain more biological insights.

Though many studies have focused on gene regulatory networks [3-6] and on signaling transduction pathways [7-9], few works have combined these two kinds of networks. Yeger-Lotem and Margalit [10] used classical graph algorithms to integrate the cellular networks of protein-protein and protein-DNA interactions. They successfully identified known simple and complex regulatory circuits and discovered many putative circuits. Yeger-Lotem *et al*. [11] searched for composite network motifs consisting of both transcription-regulation and protein-protein interactions that recur significantly more often than in random networks. Mazurie *et al*. [12] investigated the integrated network comprising transcriptional and protein-protein interaction data. They compared network motifs for different species and showed that the network motifs are not subject to any particular evolutionary pressure to preserve the corresponding interaction patterns. Recently, researchers also have integrated protein-protein and protein-DNA interactions to infer signaling-regulatory pathways, but they focused only on the pathways that explain gene expression changes in response to the knockout genes [13]. Although these works successfully extracted some characteristics of the integrated cellular network, they did not propose a method to construct the integrated cellular network of transcription regulations and protein-protein interactions under all kinds of conditions.

Many compelling biological questions center on how interactions among genes and proteins give rise to specific cellular functions. Genetic and high throughput methods have successfully identified many circuit components and their interactions. However, to explain the cellular function, there still are deficiencies, such as the incompleteness of the interactions among genes/proteins and the lack of consideration of different cellular conditions [14,15]. To address this problem, we here propose a method to construct an integrated cellular network, which can be presented under all kinds of cellular conditions, based on coupling dynamic models. A dynamic model, which is typically represented by difference equations or differential equations, is often used in many fields to describe a complex and kinetic system. Recently, systems biology and computational biology methods have widely employed the dynamic model to describe the biological functions from the dynamic system point of view [3,16,17]. The advantage of using dynamic models to construct an integrated cellular network is not only that it can provide quantitative descriptions of the network, but it also can predict the behavior of the network under different conditions, i.e., gene knockout, treatment with an external agent, etc [18].

**Figure 1:**
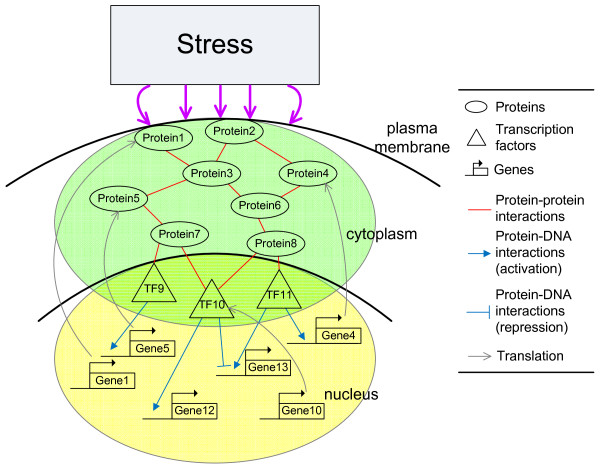
**Schematic diagram of the integrated cellular network**. The integrated cellular network consists of two subnetworks. One is the signaling regulatory pathway (the green part in the figure) in which protein-protein interactions are presented and the other is the gene regulatory network (the yellow part in the figure) in which transcription regulations exist. The transcription factors serve as the interface between the signaling regulatory pathway and the gene regulatory network.

In this article, we integrated omics data, including gene expression profiles [19], genome-wide location data [20], protein-protein interactions, and database information from SGD http://www.yeastgenome.org/[21], YEASTRACT http://www.yeastract.com/[22], BioGRID http://www.thebiogrid.org/[23], and Gene Ontology http://www.geneontology.org/[24] to construct an integrated cellular network of transcription regulations and protein-protein interactions. The schematic diagram of the integrated cellular network is shown in Figure 1. The gene regulatory network (the yellow part in Figure 1) and the signaling regulatory pathway (the green part in Figure 1) were constructed respectively, and then these two networks were combined by the interface of transcription factors (the triangles in Figure 1), which coupled two networks. Like the operating system of a computer, which is the interface between hardware and software, transcription factors (TFs) serve as the interface to interconnect gene regulatory network and signaling regulatory pathway. However, since the integrated cellular network is constructed by omics data, which are collected under different experimental conditions (some are gathered *in vitro*), it is necessary to prune these possible interactions and regulations of proteins and genes, some of which may not exist in a real-world biological organism. In this situation, it is more appealing to evaluate these interactions and regulations for a real organism under a specific condition by microarray data via dynamic interaction and regulation model. The significant or nonexistent interactions and regulations can be detected by the time profiles of microarray data through dynamic interaction and regulation model and can be pruned to construct the integrated cellular network.

Two coupling dynamic models were given in this study to describe the interplay of gene regulatory network and signaling regulatory pathway. The Akaike Information Criterion (AIC) and the *p*-value statistical method [25-27] were used to determine the significant interactions via microarray data. By the interplay of the transcription regulations and protein-protein interactions, the cellular machinery can be elucidated comprehensively. The stress response mechanism is the most adequate example to demonstrate the integrated cellular network since signaling regulatory pathway sense and transmit the stress information quickly to the corresponding TFs to activate gene regulatory network, encoding protection proteins to respond to the stresses. Therefore, the proposed method was applied to yeast *Saccharomyces cerevisiae *to investigate the integrated mechanisms of stress responses.

Living organisms have evolved complex mechanisms to respond to changes in different environmental conditions, even for the unicellular organism yeast *Saccharomyces cerevisiae *[19,28]. Such environmental changes, commonly termed as "stress", are harmful or even lethal to the survival of the cells, especially in microorganisms whose environment is highly variable. The responses to these stresses require complex networks of sensing and signal transduction pathways leading to adaptations of cell growth and proliferation as well as to adjustments of the gene expression program, metabolic activities, and other features of the cell [28]. Several regulatory systems have been implicated in modulating these responses, but the complete network of regulators of stress responses and the details of their actions, including the signals that activate them and the downstream targets they regulated, remain to be elucidated [19]. Consequently, these regulatory systems in response to environmental stresses are very suitable for the topic of integrated cellular network of transcription regulations and protein-protein interactions. In the microarray contributed by Gasch *et al*. [19], they identified the yeast stress responsive genes including stress-specific scheme and the common response to all of the stress conditions, termed the "environmental stress response" (ESR). However, they failed to determine the regulatory interactions between these genes and the dynamic characteristic of the system. In this paper, from the network or system point of view, we focused on yeast stress response and applied the proposed method to different kinds of stress (hyperosmotic stress, heat shock stress, oxidative stress), illustrating the integrated cellular network of yeast stress responses in which transcription regulations (gene regulatory network) and protein-protein interactions (signaling regulatory pathway) are integrated. Furthermore, the crosstalks of yeast stress responses were analyzed, specifying the significance of some transcription factors to serve as the decision-making devices at the center of the bow-tie structure and the crucial role for rapid adaptation scheme to respond to stress.

## Results

### Construction of integrated cellular network

**Figure 2:**
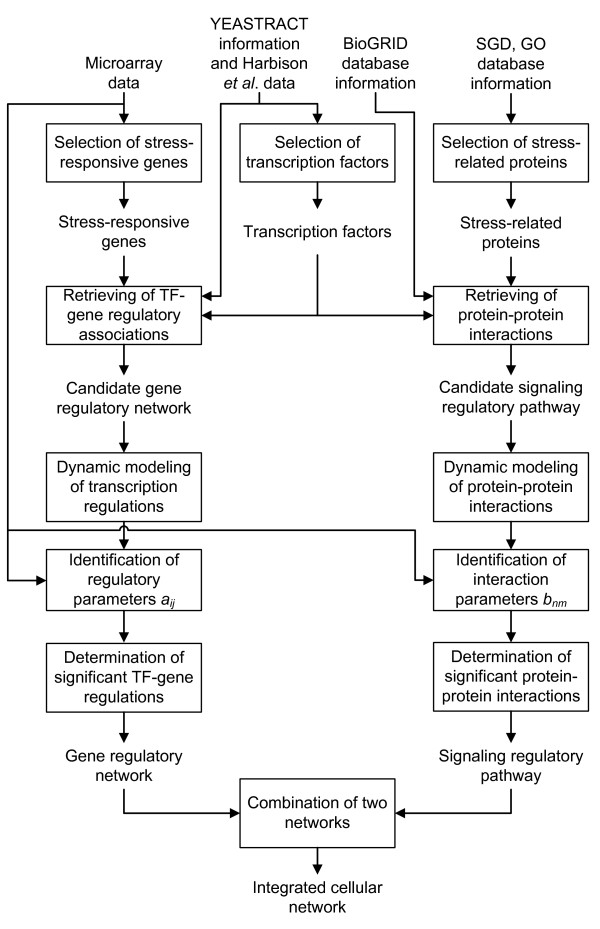
**Flowchart of the proposed method of integrated cellular network**.

Our goal is to construct the integrated cellular networks in which transcription regulations and protein-protein interactions are integrated. The flowchart of the proposed method is shown in Figure 2. Several kinds of omics data and database information were integrated, including microarray gene expression data, regulatory associations between TFs and genes, and protein-protein interaction data, as the input for the proposed method. From these data, the candidate gene regulatory network and the candidate signaling regulatory pathway, which are the rough TF-gene regulation pool and the rough protein interaction pool under all kinds of conditions, were retrieved, respectively. In this study, microarray data with time profiles was used to validate these TF-gene regulations and protein interactions integrated by these omics data. Therefore, dynamic models were used to prune the candidate gene regulatory network and the candidate signaling regulatory pathway to construct the integrated cellular network via microarray data under the specific condition of interest.

In the transcription regulation subnetwork of the integrated cellular network, the candidate gene regulatory network was depicted as a system in which TFs are inputs and target genes are outputs (see Figure 1). For a target gene *i *in the candidate gene regulatory network, the dynamic model of the gene is described by the following stochastic discrete dynamic equation(1)

where *x*_*i*_[*t*] represents the mRNA expression level of target gene *i *at time *t*, *a*_*ij *_denotes the regulatory ability of the *j*-th TF to the *i*-th target gene with a positive sign indicating activation and a negative sign indicating repression, *z*_*j*_[*t*] represents the regulation function of *j*-th TF binding to the target gene *i, λ*_*i *_indicates the degradation effect of the present time *t *on the next time *t*+1, *k*_*i *_represents the basal level, *ε*_*i*_[*t*] denotes the stochastic noise due to the model uncertainty and the fluctuation of the microarray data of the target gene. It has been shown that TF binding usually affects gene expression in a nonlinear fashion: below some level it has no effect, while above a certain level the effect may become saturated [29,30]. Thus, the regulation function *z*_*j*_[*t*] was modeled as the sigmoid function, which is one kind of Hill function, of *y*_*j*_[*t*] (the protein activity profiles of transcription factor *j*) [3,16,30-33](2)

where *f*_*j *_denotes the sigmoid function, *μ*_*j *_and *σ*_*j *_represent the mean and deviation of protein activity level of the TF *j*. The biological meaning of the equation (1) is that the mRNA expression *x*_*i*_[*t*+1] of the target gene *i *at the next time *t*+1 is determined by the present mRNA expression *x*_*i*_[*t*] plus the mRNA expression due to the present transcription regulation of *N *TFs binding to the target gene at time *t*, minus the mRNA due to the degradation of the present time, plus the basal level of mRNA expression, and some stochastic noises due to measurement noises and some random fluctuations. Because of computational simplicity, the cooperative interaction between TFs is not included in the model [16].

In the protein-protein interaction subnetwork, the candidate signaling regulatory pathway was depicted as a system in which proteins and TFs are inputs and outputs of the system, respectively (see Figure 1). For a target protein *n *in the candidate signaling regulatory pathway, the dynamic model of the protein activity is as follows(3)

where *y*_*n*_[*t*] represents the protein activity level at time *t *of the target protein *n, b*_*nm *_denotes the interaction ability of the *m*-th interactive protein to *n*-th target protein, *y*_*m*_[*t*] represents the protein activity level of *m*-th protein interacting with the target protein *n, α*_*n *_denotes the translation effect from mRNA to protein, *x*_*n*_[*t*] represents the mRNA expression level of the corresponding target protein *n, β*_*n *_indicates the degradation effect of the protein, *h*_*n *_represents the basal activity level, and *ω*_*n*_[*t*] is the stochastic noise. The rate of protein-protein interaction is proportional to the product of two proteins' concentrations [30], i.e., proportional to the probability of molecular collisions between two proteins, thus the interaction was modeled as the nonlinear multiplication scheme. For example, in the signaling transduction pathways, the phosphorylation of *y*_*n*_[*t*] by kinase *y*_*m*_[*t*] is proportional to the concentration of kinase *y*_*m*_[*t*] times the concentration of its substrate *y*_*n*_[*t*] [30]. The biological meaning of the equation (3) is that the protein activity of the target protein *n *at the next time *t*+1 is contributed by the present protein activity, plus the regulatory interactions with *M *interactive proteins, plus the translation effect from mRNA, minus the protein degradation of the present state, plus basal protein level from other sources beyond the *M *interactive proteins in the cell, and some stochastic noises. Because of the undirected nature of protein interactions, there is no direction between interacting proteins in the protein-protein interaction subnetwork.

The interactions or couplings among genes and proteins are described in the following. Some TFs *y*_*j*_[*t*] at the end of signaling regulatory pathway regulate their target genes through the regulation function *z*_*j*_[*t*] = *f*_*j*_(*y*_*j*_[*t*]) in equation (1) and then the regulated genes influence their corresponding proteins through translation effect *α*_*n*_*x*_*n*_[*t*] in equation(3). The interplay between genes and proteins can be seen from their coupling dynamic equations (1), (3) and Figure 1, in which TFs serve as the interface between the signaling regulatory pathway and gene regulatory network. In other words, the interplay of transcription regulations and protein-protein interactions constitutes the framework of the integrated cellular network.

Based on the above dynamic models in (1) and (3), the candidate gene regulatory network can be linked through the regulatory parameters *a*_*ij *_in (1) between genes and their possible regulatory TFs, and the candidate signaling regulatory pathway can be linked through the interaction parameters *b*_*nm *_in (3) between possible interactive proteins. Since the candidate gene regulatory network and candidate signaling regulatory pathway only propose possible TF-gene regulations and possible protein interactions in omics data, their reality should be confirmed by microarray data, i.e., the values of *a*_*ij *_in (1) and *b*_*nm *_in (3) should be identified and validated by microarray data. The regulatory parameters were identified via microarray data by solving the constrained least square parameter estimation problem (see Methods). The strategy was to identify the regulatory parameters *a*_*ij *_gene by gene (interaction parameters *b*_*nm *_protein by protein), so that the network identification process was not limited by the number of target genes (proteins). In other words, when identifying the regulatory parameters in candidate gene regulatory network, the regulatory parameters in equation (1) were first identified for target gene *i *and then for target genes *i*+1, *i*+2, etc. By means of model selection method such as Akaike Information Criterion (AIC) and statistical assessment like student's t-test [25-27], the significant regulations and interactions between genes and proteins were detected (see Methods). In this way, the candidate gene regulatory network and the candidate signaling regulatory pathway were pruned by microarray data, leading to construction of the gene regulatory network and signaling regulatory pathway, respectively. Based on the interface between the gene regulatory network and the signaling regulatory pathway, i.e., the transcription factors, these two networks were coupled to become the integrated cellular network, which is the output of the proposed method (see Methods). The details of each step of the algorithm are shown in Methods.

### Validation of the feasibility of the proposed method

Before applying the proposed method to construct the global view of the integrated cellular network under stress condition, we first validated the feasibility of the proposed method. While construction of gene regulatory networks using dynamic model has been successfully shown to characterize the biological system from a system point of view [3,16,17,31,32,34], to the best of our knowledge, no research has used dynamic model to infer a signaling regulatory pathway. Thus, we focused on validating the protein interaction subnetwork of the integrated cellular network.

**Figure 3:**
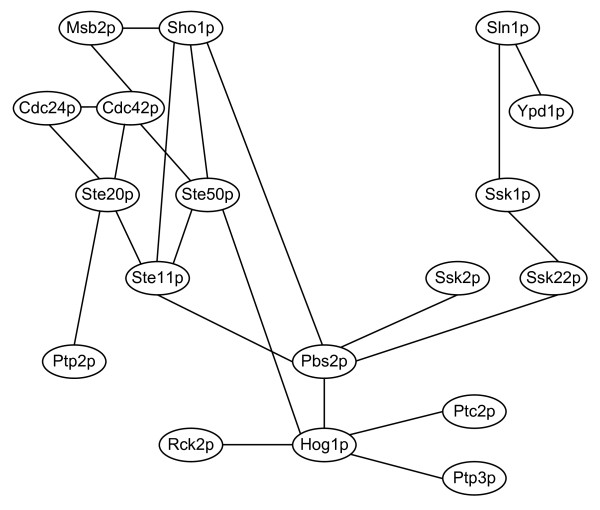
**The constructed subnetwork of HOG MAPK signaling regulatory pathway**.

We tended to reconstruct the well-studied MAPK pathway under hyperosmotic stress to validate the feasibility of the proposed method. MAPK (mitogen activated protein kinase) pathways are highly conserved eukaryotic signaling modules. The yeast MAPK pathways are involved in pheromone response, filamentous growth, osmotic response, and maintenance of cell wall integrity [28,35]. These pathways are activated by both extracellular and intracellular signals and characterized by a core cascade of MAP kinases that activate each other through sequential binding and phosphorylation reactions [28]. The osmotic activated HOG (high osmolarity glycerol) MAPK pathway, which is used by *S. cerevisiae *and other fungi to sense the osmotic pressure in the environment and maintain water homeostasis, is among the most thoroughly studied networks in yeast and is therefore an excellent benchmark against which to validate the proposed method [36,37]. Consequently, the core HOG MAPK proteins were selected and the protein interaction subnetwork among these proteins was reconstructed, which is shown in Figure 3. The reconstructed subnetwork was then investigated to see if it is consistent with the phenomena that are observed experimentally.

From the constructed subnetwork shown in Figure 3, we can find that there are two independent branches originating from Sho1p and Sln1p and converging to Pbs2p. This conforms to the observation made by Maeda *et al*. [38], who demonstrated that Pbs2p is activated by two independent signals that emanate from distinct cell-surface osmosensors by mutation analysis. In the Sln1p branch, Posas *et al*. showed that HOG MAPK cascade is regulated by a multistep phosphorelay mechanism in the Sln1p-Ypd1p-Ssk1p two component osmosensor [39]. The phosphate group is transferred sequentially from Sln1p-His576 to Sln1p-Asp1144, then to Ypd1p-His64, and finally to Ssk1p-Asp554 [39]. Posas and Saito also indicated that the Ssk2p/Ssk22p MAPKKKs are activated by the same two component osmosensor upon hyperosmotic treatment [40]. Phosphorylated Ssk2p then activates the MAPKK Pbs2p [38,40,41]. Activated Pbs2p can phosphorylate and activate Hog1p [42]. Among all these interactions observed by experiments, all were uncovered in the constructed subnetwork except for the Ypd1p-Ssk1p and Ssk1p-Ssk2p, which can be regarded as false negatives of the constructed subnetwork.

In the Sho1p branch, under hyperosmotic shock, Sho1p binds to Pbs2p and thereby recruits it to the cell surface [43]. This event may mark the generation of the protein signaling complex that recruits the MAPKKK Ste11p together with proteins required for Ste11p activation such as Cdc42p [43,44], Ste20p [44], Ste50p [45]. The interaction of Sho1p and Pbs2p and the interactions among the protein complex were identified in the constructed subnetwork. The assembly of the appropriate protein signaling complex then leads to activation of Ste20p, phosphorylation of Ste11p and subsequently phosphorylation of Pbs2p [28]. The interactions Ste20p-Ste11p, Ste11p-Pbs2p were also recognized. In yeast, the GTPase Cdc42p, together with its GTP exchange factor Cdc24p and its target Ste20p, is required to establish cell polarity during the cell cycle and is involved in cellular responses to mating pheromone and to nutritional limitation [44]. The interactions between Cdc24p, Cdc42p, and Ste20p were uncovered in the constructed subnetwork, indicating that these proteins may also be required under hyperosmotic stress.

In addition to the well-described HOG MAPK pathway osmosensors Sho1p and Sln1p, the existence of other inputs into the HOG pathway has been suggested. O'Rourke and Herskowitz identified the Msb2p protein as a weak but physiologically relevant osmosensing components in *S. cerevisiae *which functions in parallel with the Shop1 branch and activate Ste11p [46]. Recently, Tatebayashi *et al*. showed that the mucin-like transmembrane proteins Msb2p and Hkr1p are the potential osmosensors for the Shop1 branch [47]. They demonstrated that hyperactive forms of Msb2p and Hkr1p can activate the HOG pathway only in the presence of Sho1p, whereas a hyperactive Sho1p mutant activates the HOG pathway in the absence of both Msb2p and Hkr1p, indicating that Msb2p and Hkr1p are the most upstream elements in the Sho1p branch [47]. Although the interaction between Hkr1p and Sho1p was not uncovered, Msb2p was identified to interact with Sho1p in the constructed subnetwork. Besides, Msb2p can affect MAPKKK Ste11p by the interactions with Cdc42p, Ste20p, and Ste50p without the involvement of Sho1p, which is consistent with the experimental observation that Msb2p, but not Hkr1p can generate an intracellular signal in a Sho1p-independent manner [47].

Pbs2p is activated by phosphorylation by any of the three MAPKKKs Ssk2p, Ssk22p, and Ste11p and then phosphorylates the MAPK Hog1p. Phosphorylation promotes a rapid nuclear concentration of Hog1p, indicating that Hog1p is the factor that establishes the connection between the cytoplasmic part of signal pathway and its nuclear response system [48,49]. In addition to nuclear translocation of the activated Hog1p, it also mediates regulatory effects outside the nucleus. For example, Hog1p regulates the activation of protein kinase Rck2p, which controls the translational efficiency. These two proteins are necessary for attenuation of protein synthesis in response to osmotic stress [50], and the interaction among them was observed in the constructed subnetwork. Several proteins influence nuclear residence of Hog1p, such as the tyrosine phosphatases Ptp2p and Ptp3p [28]. Mattison and Ota showed that Ptp2p is a nuclear tether for Hog1p and Ptp3p is a cytoplasmic anchor, thus modulating the subcellular localization of Hog1p [51]. The Hog1p-Ptp3p interaction was indicated in the constructed subnetwork whereas Hog1p-Ptp2p was absent. Besides the absence of Hog1p-Ptp2p interaction, Ste20p-Ptp2p interaction was recognized. Although Ptp2p has been shown to be a substrate of kinase Ste20p [52], no literature evidence correlates the interaction with HOG MAPK pathway until now. Thus, further investigation is needed to clarify the Ste20p-Ptp2p interaction. Three protein phosphatases Ptc1p, Ptc2p, and Ptc3p, inactivate the HOG MAPK pathway by acting on Hog1p [53,54], however, only Ptc2p was detected to interact with Hog1p.

In summary, the constructed protein-protein interaction subnetwork was compared with the well-studied HOG MAPK pathway under osmotic stress to validate the feasibility of the proposed method. The comparison demonstrated that the well-studied MAPK pathway was mostly uncovered by the proposed method. However, there still were some interactions validated by literature evidences absent in the constructed subnetwork. The misidentification of the interaction may be because the protein activity levels were substituted by the gene expression levels (see Methods). The sensitivity of the HOG MAPK pathway is 78.57%, which shows that the signaling regulatory pathway can be successfully reconstructed by the proposed method, thus validating the feasibility of the proposed method. From the feasibility of the protein interaction network reconstruction along with the previous success of gene regulatory network characterization, we believe that the proposed method can be used to construct the integrated cellular network from the network or system point of view.

### Global view of the yeast integrated cellular network under hyperosmotic stress

The proposed method was applied to yeast *Saccharomyces cerevisiae *hyperosmotic stress, illustrating the global view of yeast integrated cellular network under hyperosmotic stress in which transcription regulations (gene regulatory network) and protein-protein interactions (signaling regulatory pathway) are integrated. Based on the microarray data from Gasch *et al*. [19], 331 hyperosmotic stress responsive genes whose transcription levels change by threefold in at least one of the time courses were identified. Among the documented TFs obtained from the YEASTRACT database and ChIP-chip data from Harbison *et al*. [20], we identified 195 TFs which have expression profiles under hyperosmotic condition. The 195 TFs, 331 genes and 4333 TF-gene regulations retrieved were regarded as candidate gene network from which we constructed the gene regulatory network of the integrated cellular network. For the protein interaction subnetwork, 102 hyperosmotic stress-related proteins were selected from GO and SGD databases. We also retrieved 714 protein-protein interactions among the 102 proteins and 195 TFs from BioGRID database to be the candidate signaling pathway to construct the signaling regulatory pathway.

**Figure 4:**
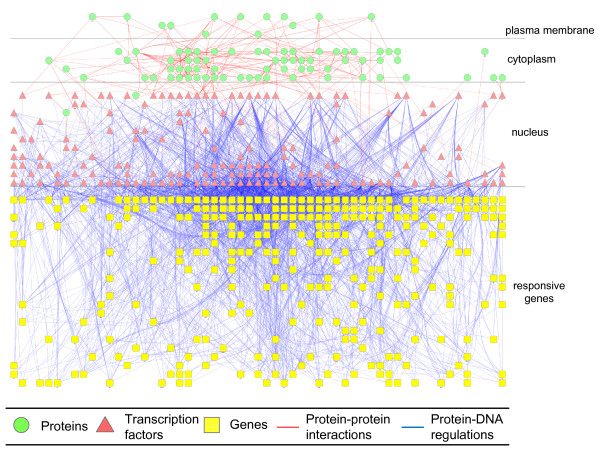
**The *S. cerevisiae *integrated cellular network under hyperosmotic stress**. The network consists of 309 genes, 162 TFs and 82 proteins, in which 2836 protein-DNA regulations and 301 protein-protein interactions are presented. The drawing is created using Cytoscape plugin Cerebral [86,87] and the subcellular compartment data is from the GO database. The directions of the regulations are omitted for simplicity.

After network construction by the dynamic modeling and identification of the significant regulations/interactions, 2836 TF-gene regulations (65.45%) and 301 protein-protein interactions (42.16%) among genes, TFs and proteins were preserved, forming the integrated cellular network under hyperosmotic stress, which is shown in Figure 4. The total preserved percentage of nodes, including all genes, TFs, and proteins, is 88.06% and the total preserved percentage of both TF-gene regulations and protein-protein interactions is 62.16%. The topology of the integrated network shows that it is a scale-free network rather than a random network. In a scale-free network, the probability that a node is highly connected is statistically more significant than in a random network, and the network's properties are often determined by a relatively small number of highly connected nodes that are known as hubs [55]. The scale-free networks are particularly resistant to random node removal but extremely sensitive to the targeted removal of hubs [56]. Therefore, the hubs are believed to be essential to improve the information transmission for network robustness and to respond quickly to protect cell function under stress [57]. In the integrated cellular network under hyperosmotic stress, in addition to the typical transcription factors such as Msn2p, Msn4p, Yap1p, Hsf1p, which regulate stress response genes, one highly connected protein, Hsp82p, and two highly connected genes, HSP12 and CTT1, were identified. Hsp82p, which belongs to Hsp90 protein family in *S. cerevisiae*, is highly conserved among eukaryotes. Experimental evidences showed that the molecular chaperone Hsp82p is required for high osmotic stress response in *S. cerevisiae *and its mutation results in osmosensitive phenotype [58]. Human Hsp90, Hsp90p, is involved in the activation of an important set of cell regulatory proteins, including many whose disregulation drives cancer. As a result, Hsp90p has been identified as important targets for anti-cancer drug development [59]. The *S. cerevisiae *gene HSP12 has been shown to be upregulated by a variety of stresses including increased temperature, osmotic stress, and glucose depletion. Under hyperosmotic stress, HSP12 is activated by the HOG pathway and is under control of Ras-PKA pathway [60]. The HSP12 encoding protein, Hsp12p, is present in the cell wall and is responsible for the flexibility of the cell wall, contributing to the resistance to osmotic stress [61]. CTT1 has been shown to be regulated by HOG pathway under osmotic induction via stress response element (STRE) and its coding protein, cytosolic catalase T, is important for survival under extreme osmotic stress [62]. Since STRE mediates the stress response not only for osmotic stress but also for heat shock, nitrogen starvation, and oxidative stress [63], CTT1 may be essential for the crosstalk of different stress responses. These results suggest that highly connected hubs, as determined by the proposed method, provide attractive targets for understanding cellular functions, performing further experiments, and even developing disease therapies [64].

### Expression profile reconstruction and prediction

**Figure 5:**
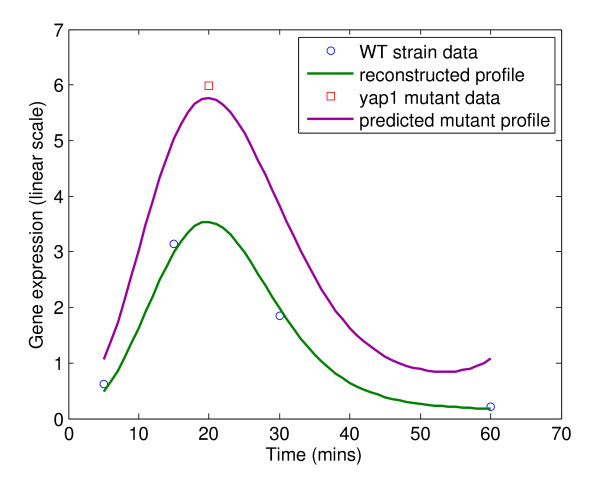
**Comparison between experimental data and reconstructed/predicted expression profile of HXT5 under heat shock**. The reconstructed expression profile for the WT strain (training data) and the predicted *yap1 *mutant profile (testing data) are computed by the dynamic model. The WT strain data and the *yap1 *mutant data under heat shock are from Gasch *et al*. [19]. The correlation coefficient between the WT strain experimental data and the reconstructed expression profile is 0.9949.

The method proposed to construct the integrated cellular network is to use expression profiles of microarray data via a dynamic model, which can provide a qualitative description of the network as well as quantitative dynamics of the network. Since Gasch *et al*. [19] previously explored genomic expression patterns in the yeast *S. cerevisiae *responding to diverse environmental transitions for almost every yeast gene, the proposed method can also be applied to other stress conditions in addition to hyperosmotic stress. Under heat shock stress, Gasch *et al*. not only determined the response of the WT strain, but also of the *yap1 *mutant strain [19], which facilitates the predictive power verification of the proposed method. By the same procedure, we first constructed the integrated cellular network under heat shock stress for the WT strain yeast. Then with the trained dynamic models for the WT strain (training data), we tended to predict the gene expression for the *yap1 *mutant strain (testing data). The testing result of gene HXT5 is shown in Figure 5. The data shown in the figure verifies that the dynamic model is useful for modeling the integrated network provided that the expression profile reconstructed by dynamic model is very similar to the original WT strain data from Gasch *et al*. [19] (correlation coefficient = 0.9949). Furthermore, comparison of the experimental data with the predicted expression profile of the *yap1 *mutant strain for HXT5 under heat shock reveals the predictive power of the proposed method. The predicted HXT5 expression from the trained dynamic model for training data is approximately the same as the experimental data (an external testing data). It can be found that predicted HXT5 expression of the *yap1 *mutant strain is more than its expression of the WT strain. This predicted result is quite reasonable since HXT5, which encodes a functional hexose transporter, is expressed during conditions of relatively slow growth rates and the expression is regulated by growth rates of cells [65,66]. Yap1p is a basic leucine zipper transcription factor that is required for many kinds of stress response mechanisms including heat shock stress. Once mutated, the heat sensitivity would be increased, causing the growth rate of the cell to decrease, thus increasing the expression of HXT5 gene. Because of the lack of more time points of mutation expression profile from Gasch *et al*. [19], we can only compare the predicted expression profile of HXT5 with the single expression data by experiment. However, the predictive power of the proposed method can be verified.

### Crosstalks of the yeast stress responses

Because of the convenience of integrated cellular network construction we proposed, it is appropriate to investigate the crosstalks of the yeast stress responses from the global network point of view. Genes/proteins responsible for hyperosmotic stress, heat shock, and oxidative stress were selected, which resulted in 1260 genes, 190 TFs, 348 proteins, 11262 TF-gene regulations, and 2276 protein-protein interactions in the candidate integrated cellular network. Then the integrated cellular networks under these stresses were constructed, respectively. Comparison of the three yeast integrated cellular networks is shown in Table 1. From the data shown in Table 1, four observations were made. (1) Protein-protein interactions are more conserved than TF-gene regulations. Comparing all three integrated cellular networks under different stresses, we can find that protein-protein interactions are more conserved than TF-gene regulations (27.11% vs. 6.51%). The observation implicates that under different stresses, the signaling regulatory pathways are more similar than the gene regulatory networks are. In other words, yeast use similar signaling regulatory pathway to sense and transmit the environmental changes but use distinct gene regulatory network to adapt themselves to different environmental conditions. (2) The crosstalks of the three integrated cellular networks could be interpreted as the core stress responses. Since under different stresses, yeast use the same subnetwork of signaling regulatory pathway/gene regulatory network, this subnetwork may be essential for the yeast stress response mechanisms and therefore can be interpreted as the core stress responses. In the core stress responses, there are some important proteins/TFs/genes which are highly connected. For example, Hsp82p and Hsc82p are two hub proteins in the signaling regulatory pathway. Hsp82p and Hsc82p, which are redundant in function and nearly identical with each other, are essential for osmotic and heat shock stresses [58,67]. They are also required for the activation of many key cellular regulatory and signaling proteins like kinases and transcription factors [68]. Consequently, it is reasonable to recognize these proteins in the crosstalks of signaling regulatory pathway. The TFs Msn2p, Yap1p, Sfp1p, which have been shown to regulate stress responsive genes, were also identified in the core stress responses. The genes SGA1, YGP1, and RPS3 are highly connected in the crosstalks of gene regulatory network, but the mechanisms in which the genes involved to adapt to environmental stresses require further investigation. (3) The TFs serve as the 'knots' in the bow-tie structure and can be viewed as the decision-making devices of the stress response mechanism. Along with the observation that protein-protein interactions are more conserved than TF-gene regulations and the fact that the gene expression program is controlled by a portion of proteins, TFs, that is smaller than that of the whole genome, the TFs can be viewed as the 'knots' in the bow-tie structure and the decision-making devices of the stress response mechanism. Sensing of stress is the first step for stress response mechanisms, activating different signaling regulatory pathways under different stress conditions. The signals transmitted into the cell will converge on the TFs to adapt different gene programs in order to respond to the environmental stresses. Our results demonstrated that 136 out of 190 TFs are conserved under all three stresses, indicating that different stress mechanisms make use of the same set of TFs. Thus, the interface between gene regulatory network and signaling regulatory pathway, TFs, serve as the 'knots' in the bow-tie structure of the stress response mechanism [69]. Because they are knots of the bow-tie structure of the stress response mechanisms, the TFs are also highly connected hubs, which improve the efficiency of information exchange, in the complex networks. Rapid information exchange has been shown to be very important for cells to survive under stress. Besides, when the cell suffers from different types of stress, the TFs receive different signals from the signaling regulatory pathway and then make decisions for condition-specific program of adaptation to respond to these stresses. Consequently, they are also viewed as the decision-making device [30]. (4) There may be cross-protection between hyperosmotic stress response and heat shock response. The crosstalks between these three stress responses were further distinguished, showing that the crosstalks between hyperosmotic stress response and heat shock response are much more than each one with oxidative stress response. The result can be attributed to the situation where hyperosmotic stress and heat shock stress frequently happen simultaneously, thus sharing part of response mechanism. This circumstance can also be observed in other organisms. For example, intertidal organisms exposed to heat shock normally also experience hyperosmotic stress at the same time [70]; plants suffering osmotic stress caused by drought in the summer often also have high-temperature stress [71]. Consequently, there may be cross-protection between hyperosmotic stress response and heat shock response, which protect the cell from the harm of these two stresses simultaneously. From the crosstalks and the network comparison of different stress responses, we can investigate the differences and the similarities of different stress response mechanisms in order to understand the cellular machinery more thoroughly [72].

**Table 1:** Comparison of the three yeast integrated cellular networks under hyperosmotic stress, heat shock, and oxidative stress.

	# Genes	# TFs	# Proteins	# TF-gene interactions	# Protein-protein interactions
**Candidate network**	1260	190	348	11262	2276
**Osmotic**	1043	176	260	6931	959
**Heat**	1161	179	280	8330	1186
**Oxidative**	884	171	250	5556	858
**Osmotic & Heat**	804	164	252	2615	873
**Heat & Oxidative**	659	161	242	2175	794
**Osmotic & Oxidative**	609	159	227	1775	658
**All three**	388	136	220	733	617

The first row indicates the statistics of the candidate integrated network, which are extracted from omics data and database information. The second to fourth rows represent the statistics for integrated network identified under each stress conditions. The fifth to seventh rows reveal the crosstalks among each two of three stress conditions and the last row shows the crosstalks among all three stress conditions.

## Discussion

Since the network reconstruction process is organism specific and is based on different datasets such as annotated genome sequence, high-throughput network-wide datasets and bibliomic data on the detailed properties of individual network components [15], it is not easy for biologists to efficiently construct the integrated cellular network by traditional genetic/molecular manipulation. By integrating different kinds of omics data including microarray gene expression data, regulatory associations between TFs and genes, and protein-protein interaction data, we have provided an efficient method based on coupling dynamic models and statistical assessments to construct an integrated cellular network in which transcription regulations (gene regulatory network) and protein-protein interactions (signaling regulatory pathway) are integrated. The stress response mechanism is the most adequate example to demonstrate the integrated cellular network because the signaling regulatory pathway is highly expressed to sense and transmit the stress information and the gene regulatory network is expressed to encode protection proteins to respond to the stress. Therefore the proposed method was applied to construct the integrated cellular network of *S. cerevisiae *under different stress conditions. Our results showed that the constructed integrated network can be validated by literature evidences, indicating the feasibility of the proposed method. The highly connected hubs which are functionally relevant to the hyperosmotic stress response were also identified and the predictive power of the proposed method was also demonstrated. Beyond hyperosmotic stress, the integrated network under heat shock and oxidative stress were constructed and the crosstalks of these networks were analyzed, which is not easily accomplished by traditional biological experiments.

In this work, a coupling dynamic model was used to characterize the yeast stress response mechanism from the network or system point of view. We provide a systematic and efficient way to move forwards studying the stress response networks rather than the stress response genes, which can be done by performing microarray experiments. However, one inconvenience of using this method is the lack of protein activity profiles. Thus there may be some inaccuracies when modeling the integrated cellular network with the substitution of gene expression levels. Even so, the well-studied HOG MAPK pathway was mostly uncovered by the proposed method and the results were validated by literature evidences. Once the genome-wide protein activity levels have been obtained, the accuracy can be improved. The advantage of the proposed method over others is its flexibility. It can be employed under all kinds of experimental conditions such as cell cycle or different kinds of diseases and for all kinds of organisms. Also, our method can be applied to identify and integrate the cellular networks of all sizes (see Results and Methods). In other words, the use of the proposed method is not limited by the number of genes/proteins. This benefit allows us to explore both a global view of the integrated network and a more detailed local view of the gene/protein regulation of interest. Since the global view of the integrated cellular network is not easily obtained by time- and labor-consuming biological experiments, the proposed method can be used by biologists to extract some useful information such as highly connected hubs which is possibly for further investigation. With slight modification, for example, by adding one clustering step before dynamic modeling, the proposed method can also be used to construct the module networks, in which sets of genes are co-regulated to respond to different conditions [73]. The major advantage of dynamic modeling is the supply of quantitative dynamics of the network rather than qualitative descriptions. Because of the gene/protein dynamic system we constructed, computational methods can easily be employed to investigate the integrated gene/protein network. For example, we can make use of the dynamic systems to simulate any kind of perturbation such as gene knockout or the interference of external disturbances to see the system response for the perturbation. This can be very useful since the integrated gene/protein system can be modified arbitrarily to see the predicted consequences. In addition, the dynamics of the system can be used for theoretical analysis of the network such as robustness analysis and the signal transduction ability analysis. The system analysis and the computational simulation can be served as the pre-screen and the guidance for further experiments in the field of synthetic biology [74]. Furthermore, the integrated cellular network can be mapped to the metabolic network to see how the cells make use of the metabolism to respond to different stresses and to unravel where and when certain parts of the network are active, elucidating the cellular machinery from a more comprehensive perspective [75,76].

## Conclusions

At present, there is no satisfactory method to construct the integrated cellular network, combining a gene regulatory network and a signaling regulatory pathway. We here provide a systematic and efficient method to integrate different kinds of omics data to construct the integrated cellular network via microarray data based on coupling dynamic models and statistical assessments. The integration of transcription regulations and protein-protein interactions gives more insight into actual biological networks and it is more predictive than those without integration [77]. The integrated network construction represents an initial step for further network comparison and analysis. In our analyses of the *S. cerevisiae *stress response mechanism, the significance of some transcription factors to serve as the decision-making devices at the center of the bow-tie structure and the crucial role for rapid adaptation scheme to respond to stress are indicated. Furthermore, we also identify some genes/proteins which are relevant to the stress responses or are attractive targets for potential disease therapies [64]. The method is shown to be powerful and flexible and can be used under different conditions and for different species. The dynamic models of the overall integrated cellular network provide a very useful tool for theoretical analyses and for further experiments in the fields of network biology and synthetic biology.

## Methods

### Data selection and preprocessing

There are three kinds of data used in the method--microarray gene expression data, regulatory associations between TFs and genes, and protein-protein interaction data. We used the genome-wide microarray data from Gasch *et al*. [19] as the stress responses data. They explored genomic expression patterns in the yeast *Saccharomyces cerevisiae *responding to diverse environmental transitions for almost every yeast gene, including temperature shocks, hyper- and hypo-osmotic shock, amino acid starvation, hydrogen peroxide shock, etc [19]. The genes whose mRNA levels changed by threefold in at least one of the time courses were chosen as the significantly responsive genes. The responsive genes with more than 30% of the time points missing, which were regarded as unreliable experimental data, were excluded from further analyses. The transcription factors and the regulatory associations between TFs and target genes were obtained from YEASRTACT database http://www.yeastract.com/ and genome-wide location (ChIP-chip) data from Harbison *et al*. [20], in which the genomic occupancy of 203 DNA-binding TFs was determined in yeast. YEASTRACT (Yeast Search for Transcriptional Regulators And Consensus Tracking) is a curated repository of more than 34469 regulatory associations between TFs and target genes in *Saccharomyces cerevisiae*, based on more than 1000 bibliographic references [22]. The significant binding of Harbison *et al*. [20] was selected as *p *< 0.001, as indicated in their paper. By the stress-responsive genes and the TF-gene regulatory associations obtained, we have the candidate gene regulatory network, which is the rough TF-gene regulation pool for building the gene regulatory network. The last dataset, protein-protein interaction data, were extracted from BioGRID database http://www.thebiogrid.org/. The Biological General Repository for Interaction Datasets (BioGRID) database was developed to house and distribute collections of protein and genetic interactions from major model organism species. BioGRID currently contains over 198000 interactions from six different species, as derived from both high-throughput studies and conventional focused studies [23]. The stress-related proteins of the candidate integrated cellular network were selected from Gene Ontology (GO) http://www.geneontology.org/ and *Saccharomyces *Genome Database (SGD) http://www.yeastgenome.org/. Again, based on the stress-related proteins and the protein-protein interactions extracted, we have the candidate signaling regulatory pathway, which provides the rough protein interaction pool for constructing the signaling regulatory pathway.

In the microarray data from Gasch *et al*. [19], there are some missing values. The cubic spline interpolation method, which employs piecewise third-order polynomials to fit data points, was used to complement the missing values [78-80]. In order to fit the dynamic model, the microarray gene expression data was transformed from log2 scale to linear scale.

### Dynamic model of the integrated cellular network

The candidate gene regulatory network and the candidate signaling regulatory pathway were constructed respectively (see Figure 2), and the stochastic discrete coupling dynamic models of these candidate networks are shown in equation (1) and equation (3). The interplay of the transcription regulations and protein-protein interactions constitutes the framework of the candidate integrated cellular network.

### Identification of the regulatory parameters a_*ij *_and interaction parameters b_*nm*_

After constructing the coupling dynamic models of the candidate integrated cellular network, the regulatory/interaction parameters in the models have to be identified using the microarray data we have. The strategy is to identify the integrated cellular work gene by gene (protein by protein). Before determining the identification method, we first examine the dynamic models carefully. In equation (1), the basal expression level *k*_*i *_should be always non-negative, since the microarray expression of the genes are always non-negative. Because the parameters in equation (1) have certain constraints, the regulatory parameters were identified by solving the constrained least square problems.

Equation (1) can be rewritten as the following regression form.(4)

where *φ*_*i*_[*t*] denotes the regression vector which can be obtained from the processing above. *θ*_*i *_is the parameter vector of the target gene *i *which is to be estimated. In order to avoid overfitting when identifying the regulatory parameters, the cubic spline method [78-80] was also used to interpolate extra time points for the gene expression data. By the cubic spline method, we can easily get the values of {*z*_*j*_[*t*_*l*_] *x*_*i*_[*t*_*l*_]} for *l *∈ {1, 2, ···, *L*} and *j *∈ {1, 2, ···, *N*}, where *L *is the number of expression time points of a target gene *i*, and *N *is the number of TFs binding to the target gene *i*. Equation (4) at different time points can be arranged as follows(5)

For simplicity, we define the notations *X*_*i*_, Φ_*i*_, and E_*i *_to represent equation (5) as follows(6)

The constrained least square parameter estimation problem is formulated as follows(7)

where *A *= [0 ··· 0 0 -1], b = 0 give the constraints to force the basal level *k*_*i *_in equation (1) to be always non-negative, i.e., *k*_*i *_≥ 0. The constrained least square problem can be solved using the active set method for quadratic programming [81,82].

Again, equation (3) can be rewritten in the following regression form.(8)

where *ψ*_*n*_[*t*] indicates the regression vector and *η*_*n *_is the parameter vector to be estimated. By cubic spline method, at different time points, equation (8) can be presented as the following equation.(9)

The identification problem is then formulated as follows(10)

where *C *= diag [0 ··· 0 -1 0 -1] and d = [0 ··· 0]^*T*^, indicating that the translation effect *α*_*n *_and the basal activity level *h*_*n *_are non-negative. Since large-scale measurement of protein activities has yet to be realized and it was demonstrated that 73% of the variance in yeast protein abundance can be explained by mRNA abundance [83], mRNA expression profiles were used to substitute for the protein activity levels when identifying the interaction parameters. Due to the lack of protein activity data and the undirected nature of the protein interactions in the candidate signaling regulatory pathway, the sign of the regulatory parameters *b*_*nm*_'s does not imply activation or repression and there is no direction between interacting proteins.

### Determination of significant interaction pairs

When the regulatory parameters were identified, Akaike Information Criterion (AIC) [25,26] and student's t-test [27], which is used to calculate the *p*-values of the regulatory/interaction abilities, were employed for both model selection and determination of significant interactions in the integrated network. The AIC, which attempts to include both the estimated residual variance and model complexity in one statistics, decreases as the residual variance decreases and increases as the number of parameters increases. As the expected residual variance decreases with increasing parameter numbers for non-adequate model complexities, there should be a minimum around the correct parameter number [25,26]. Therefore, AIC can be used to select model structure based on the regulatory abilities and the interaction abilities (*a*_*ij*_'s, *b*_*nm*_'s) identified above. Due to computational efficiency, it is impractical to compute the AIC statistics for all possible regression models. Stepwise methods such as forward selection method and backward elimination method are developed to avoid the complexity of exhaustive search [84,85]. However, in the case of backward selection method, a variable once eliminated can never be reintroduced into the model, and in the case of forward selection, once included can never be removed [85]. Thus, the stepwise regression method which combines forward selection method and backward elimination method was applied to compute the AIC statistics. Once the estimated regulatory/interaction parameters were examined using the AIC model selection criteria, student's t-test was employed to calculate the *p*-values [27] to determine the significant regulations/interactions. The *p*-values computed were then adjusted by Bonferroni correction to avoid a lot of spurious positives [27]. The regulations/interactions which adjusted *p*-value ≦ 0.05 were determined as significant regulations/interactions and preserved in the integrated cellular network.

### Combination of gene regulatory network with signaling regulatory pathway

Once the gene regulatory network and signaling regulatory pathway were modeled and identified separately, there were two sets of dynamic equations comprising respective networks. Since there are common components to couple these two networks - transcription factors, these equations can simply be merged to become one system, the integrated cellular network. In the schematic diagram of the integrated cellular network in Figure 1, for example, Gene13 was modeled as

when the gene regulatory network was identified. Also, TF10 was modeled as

when constructing the signaling regulatory pathway. In these dynamic equations, since there are common components *y*_TF10_[*t*] coupling these two networks, these two equations were simply merged to become one system

which described one part of the integrated cellular network in Figure 1.

## Authors' contributions

YCW developed the method, performed the simulation, evaluated the results and wrote the manuscript. BSC conceived of the study, provided essential guidance and revised the manuscript. All authors read and approved the final manuscript.
